# Selected Lark Mitochondrial Genomes Provide Insights into the Evolution of Second Control Region with Tandem Repeats in Alaudidae (Aves, Passeriformes)

**DOI:** 10.3390/life14070881

**Published:** 2024-07-15

**Authors:** Chuan Jiang, Hui Kang, Yang Zhou, Wenwen Zhu, Xilong Zhao, Nassoro Mohamed, Bo Li

**Affiliations:** 1 College of Wildlife and Protected Area, Northeast Forestry University, Harbin 150040, China; 1121672544@nefu.edu.cn (C.J.); kanghui@ihb.ac.cn (H.K.); zhaoxilong@nefu.edu.cn (X.Z.); nassoro.ali@mwekawildlife.ac.tz (N.M.); 2 BGI Research, Shenzhen 518083, China; zhouyang@genomics.cn; 3 BGI Research, Wuhan 430074, China; 4 School of Life Sciences, Heilongjiang University, Harbin 150080, China; 2222556@s.hlju.edu.cn; 5 State Forestry and Grassland Administration Detecting Center of Wildlife, Harbin 150040, China

**Keywords:** Alaudidae, mitochondrial genome, phylogeny, gene rearrangement, tandem repeat, slipped-strand mispairing, turnover model

## Abstract

The control region (*CR*) regulates the replication and transcription of the mitochondrial genome (mitogenome). Some avian mitogenomes possess two *CRs*, and the second control region (*CR2*) may enhance replication and transcription; however, the *CR2* in lark mitogenome appears to be undergoing loss and is accompanied by tandem repeats. Here, we characterized six lark mitogenomes from *Alaudala cheleensis*, *Eremophila alpestris*, *Alauda razae*, and *Calandrella cinerea* and reconstructed the phylogeny of Passerida. Through further comparative analysis among larks, we traced the evolutionary process of *CR2*. The mitochondrial gene orders were conserved in all published lark mitogenomes, with *Cytb-trnT-CR1-trnP-ND6-trnE-remnant CR2* with tandem repeat-*trnF-rrnS*. Phylogenetic analysis revealed Alaudidae and Panuridae are sister groups at the base of Sylvioidea, and sporadic losses of *CR2* may occur in their common ancestor. *CR* sequence and phylogeny analysis indicated *CR2* tandem repeats were generated within *CR2*, originating in the ancestor of all larks, rather than inherited from *CR1*. The secondary structure comparison of tandem repeat units within and between species suggested slipped-strand mispairing and DNA turnover as suitable models for explaining the origin and evolution of these repeats. This study reveals the evolutionary process of the *CR2* containing tandem repeat in Alaudidae, providing reference for understanding the evolutionary characteristics and dynamics of tandem repeats.

## 1. Introduction

The mitochondrial (mt) genome (mitogenome) typically contains thirteen protein-coding genes (PCGs), two ribosomal RNAs (*rRNA*), twenty-two transfer RNAs (*tRNA*), and one non-coding region (control region, *CR*) in most vertebrate animals [1]. Since the complete mitogenome of the domestic chicken (*Gallus gallus*) was sequenced, gene rearrangements have been identified frequently in many bird mitogenomes, and seven alternative mt gene orders have been reported in hotspot regions of *CR* and flanking genes [2,3,4,5,6,7,8,9,10,11,12]. These rearranged gene orders can be derived from a common ancestor by a tandem duplication of *CR* and neighboring genes followed by subsequent degeneration and/or loss of partial duplicate genes, i.e., tandem duplication and random loss (TDRL) model [10,11,12,13]. Moreover, intermolecular recombination has also been proposed as a mechanism underlying the generation of the aforementioned mt gene orders [14].

As the region of the mitogenome with the highest rate of evolution, *CR* generally controls the transcription of mt genes and H strand replication. Some bird lineages have two copies of *CRs* in their mitogenomes along with gene rearrangements [2,3,4,5,6,7,8,9,10,11,12]. This duplication might be linked to a higher metabolic rate [11] and potentially even longer lifespans [15]. The second *CR* (*CR2*) can maintain high similarity with *CR1* through concerted evolution [4,5] or randomly lose some sequences to become remnant *CR2* (*rCR2*) and eventually be lost [9]. Tandem repeats have been widely observed in the *CR* of animal mitogenome, and the number of repeat units can vary among species [16], populations [17], or even within an individual [18], resulting in highly variable lengths of the *CR*. Tandem repeat can be present in both *CR1* and *CR2*, but it is still unknown whether the tandem repeats in *CR2* originated from *CR1*. Several models have been proposed to interpret the origin and evolution of tandem repeats, including recombination and transposition [19], unequal crossing over [20], and slipped-strand mispairing [21,22]. However, there is still a limited understanding of the evolutionary characteristics of tandem repeats.

The lark family Alaudidae comprises 21 genera and 100 species distributed across six continents [23,24,25]. Previous research has found that Alaudidae mitogenomes have an integral *CR1* and one *rCR2* with tandem repeats [26,27,28]. Recently, advancements have been made in exploring possible scenarios and mechanisms for the evolution of duplicated regions within mitogenomes of Passeriformes [11,12]. Our study contributes to this growing body of work by investigating the evolutionary progression of gene arrangement and *rCR2* in Alaudidae.

On the other hand, it was suggested that mapping mt gene orders onto the resolved phylogenetic tree using mitogenomes was useful for understanding the evolutionary progression of gene arrangement and duplicated *CR* in Alaudidae [11]. However, the phylogeny of Alaudidae, particularly the interfamily relationships, remains debated. Several studies revealed that Alaudidae was sister to the clade of other Sylvioidea species [11,28,29,30,31,32,33,34,35,36], while the other showed that Alaudidae has a close relationship with Panuridae [37,38,39,40,41,42,43,44,45].

To address the above questions, we amplified and sequenced four mitogenomes of *Alaudala cheleensis* and *Eremophila alpestris* and assembled two mitogenomes of *Alauda razae* and *Calandrella cinerea* using sequencing data obtained from the SRA database. We then characterized their structure by comparing them with other published Alaudidae mitogenomes. Using Sylvioidea mitogenomes available in GenBank up to April 2024, we reconstructed the phylogeny of Passerida and mapped the mt gene order and tandem repeats onto the phylogenetic tree. With the use of *CR* sequence analysis, we focused on mt gene arrangement and duplications of *CR* and tandem repeats in Alaudidae and attempted to reconstruct their evolutionary progression.

## 2. Materials and Methods

### 2.1. Samples Collection, DNA Extraction, and Data Download

We collected muscle samples of two *A. cheleensis* and two *E. alpestris* individuals in Manzhouli, Inner Mongolia Autonomous Region. These specimens were stored at −80 °C and were provided by the sample library of the State Forestry and Grassland Administration Detecting Center of Wildlife (Harbin, China). Samples were legally collected and were properly preserved for applications in law enforcement mainly in cases involving forensic analysis. Sample collection and experimentation were approved by the Northeast Forestry University Institutional Review Board of Ethics and Administration of Experimental Animals.

Total genomic DNA was isolated using a tissue extraction kit (AxyPrep DNA, Hangzhou, China) and quantified with a DU-640 Nucleic Acid-Protein Analysis System (Beckman Coulter, Brea, CA, USA) according to the user’s manual.

Sixty-one Passeriformes mitogenomes including six larks and two Psittaciformes mitogenomes were downloaded from NCBI for phylogenetic analysis (detail see Section 2.4). In addition, based on the sampling location of an *A. cheleensi* sample (44.10 N 100.93 E) and a phylogenetic analysis of some subspecies, we identified an unpublished mitogenome (MN356181) belonging to the subspecies *A. cheleensi heinei* (Figure S1). As a note, this subspecies is currently recognized as the independent species *A. heinei* [46,47], and we adopted this classification in the present study. The raw sequencing data of *Alauda razae* (SRX7050284) and *Calandrella cinerea* (SRX16766684) [48] were downloaded from the SRA database for subsequent mitogenome assembly.

### 2.2. PCR Amplification and Sequencing

To minimize the possibility of obtaining nuclear copies of mt genes (Numts), the entire mitogenomes were amplified in long overlapping fragments using the long and accurate polymerase chain reaction (LA-PCR) by nine primer pairs (Table S1). LA-PCRs were conducted in an Eppendorf thermocycler in a volume of 50 μL containing approximately 100 ng DNA template, 1× PCR buffer (10 mM Tris–HCl pH 8.3, 50 mM KCl, and 1.5 mM MgCl_2_), 0.2 mM dNTP, 0.2 μM each primer, and 2.5 U LA Taq DNA polymerase or PrimeSTAR GXL DNA Polymerase (TaKaRa, Dalian, China). Thermal cycling included 94 °C for 1 min, followed by 35 cycles of 94 °C for 1 min, 50–60 °C for 45 s, 72 °C for 1 min 30 s, and a final extension of 10 min at 72 °C. Amplification products were separated with a 1% agarose gel. The bands containing exact DNA fragments from the same individual were recovered and purified using an AxyPrep™ DNA Gel Extraction Kit (AxyGen, Hangzhou, China) according to the manufacturer’s instructions. Recovered PCR products were sequenced directly using Sanger sequencing in an ABI 3730 DNA Analyzer following the primer-walking strategy (performed by BGI, Beijing, China).

### 2.3. Mitogenome Assembly, Annotation, and Mitogenome Analysis

The Sanger sequencing data of *A. cheleensis* and *E. alpestris* were assembled using the SeqMan software (DNAStar, Madison, WI, USA) based on overlapping fragments. For the Illumina sequencing data of *A. razae* and *C. cinerea*, quality control was initially conducted using fastp with default parameters [49]. Subsequently, the assembly was performed to reconstruct mitogenomes using GetOrganelle pipeline [50], with the *A. arvensis* mitogenome used as a reference [26]. Genes in the mitogenomes were annotated using Geneious 10.1.3 [51], employing other Alaudidae species as references [26,27,28]. The annotation of tRNA genes referred to the results of tRNAscan-SE 1.21 [52]. Annotation of all mt genes was checked by aligning them with those of other Passeriformes species from GenBank.

The circular mitogenome maps were drawn using the online tool CGView [53]. The nucleotide composition was calculated and analyzed using Geneious 10.1.3 [51]. Composition skew values were calculated using AT-skew = [A − T]/[A + T] and GC-skew = [G − C]/[G + C] [54]. The relative synonymous codon usage (RSCU) was analyzed using Phylosuit v.1.2.3 [55]. The secondary structures of RNAs were predicted using MITOS [56]. The conserved elements in *CRs* were analyzed with reference to previous studies [26,57,58], and the tandem repeats in *CRs* were detected by Tandem Repeat Finder [59]. Potential secondary structures of tandem repeat unit were examined, and their free energies were estimated using MFOLD [60]. The local alignment of all sequences was calculated using the program water from the EMBOSS package [61] based on the Smith–Waterman algorithm. To better trace the evolutionary history of tandem repeats in *CR2*, we examined the presence of tandem repeats in the *CRs* of all published Sylvioidea mitogenomes (Table S2) and performed a Fisher’s exact test to examine the association between the presence of tandem repeats in *CR1* and *CR2*.

### 2.4. Phylogenetic Analysis

Based on the above inspection results (Table S2), we sampled all types of mitogenomes (categorized by whether *CR2* is complete and whether *CRs* has tandem repeats) within each family under Sylvioidea for phylogenetic analysis. In addition, we also included 17 species from the Muscicapoidea, Passeroidea, and Paroidea as well as 2 species from the Psittaciformes as outgroups, resulting in a total of 71 mitogenomes for phylogenetic analysis (Table S3).

To maximize the phylogenetic information, all *RNAs* and *PCGs* [62] were selected for all species except for two species (*Oxylabes madagascariensis* and *Donacobius atricapilla*) due to missing *ND6*, *trnP*, and *trnE*. *CRs* were excluded from phylogenetic analyses since these sequences had poor phylogenetic performance and might produce tree topologies inconsistent with real evolutionary relationships among species [11].

We used Phylosuit v.1.2.3 [55,63] to conduct, manage, and streamline the analyses with the help of several plug-in programs. The PCGs and *RNAs* sequences were aligned in MAFFT with the default parameters [64], and the PCG alignments were further refined using the codon-aware program MACSE v2.06 [65]. The sites suitable for phylogenetic analysis were selected in Gblocks [66] and were then concatenated by the plug-in concatenate sequence option in PhyloSuite v.1.2.3. The optimal partitioning scheme for nucleotide substitution models for each gene was determined by PartitionFinder v.2.0 [67] under a greedy search algorithm with linked branch lengths based on AICc. Table S4 lists the best-fit substitution models and partitioning schemes for each gene.

Phylogenetic analyses were conducted using BI and ML methods. The BI analysis was performed using MrBayes v.3.1.2 [68] based on the optimal model of each partition. Two sets of four chains were allowed to run simultaneously for 80,000,000 generations, and each set was sampled every 1000 generations. The convergence and mixing of the chains of each analysis were evaluated using Tracer v.1.6.1 [69] to check that the ESS values were all superior to 200. A consensus tree was then calculated after excluding the first 25% of trees as burn-in. The ML analysis was performed using IQ-TREE v.1.6.8 [70] under the models selected for each identified partition and SH-aLRT assuming 10,000 replicates and nonparametric bootstrapping with 1000 replicates were used to estimate the node reliability.

## 3. Results

### 3.1. General Characteristics of Six Mitochondrial Genomes

**Figure 1:**
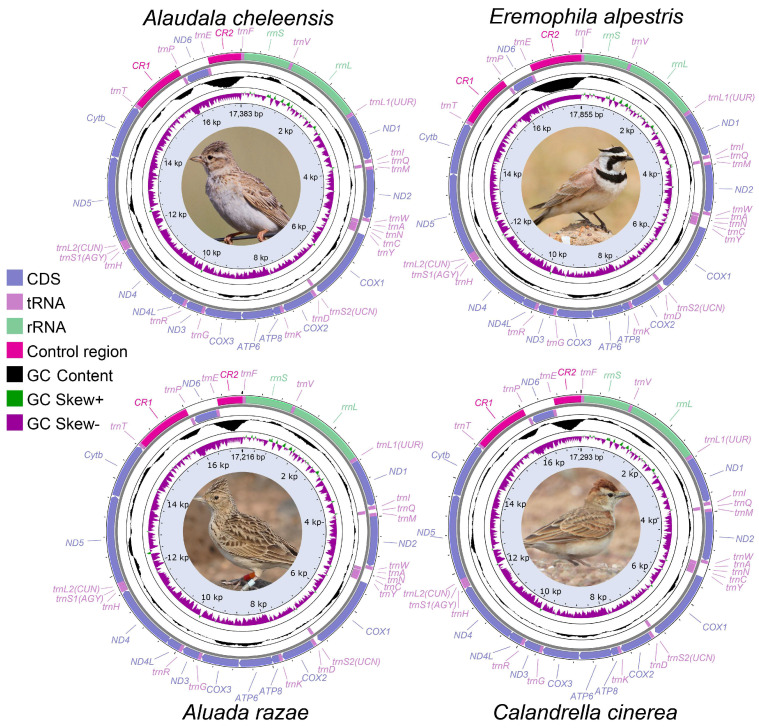
Mitochondrial genome map of four lark species. Genes encoded on the H or L strand are indicated on the outside or inside of the circular mitogenome map, respectively. Arrows indicate the gene transcription direction. The GC skew (50 bp window size) is plotted using a green and purple sliding window, indicating positive and negative values, respectively. GC content (500 bp window size) shows a deviation from the average GC content of the entire sequence. The photo is sourced from Birds of the World [71].

The sizes of the mitogenomes for two *A. cheleensis*, two *E. alpestris*, *A. arazae*, and *C. cinerea* were found to be 17,383 bp, 17,383 bp, 17,855 bp, 17,702 bp, 17,216 bp, and 17,293 bp, respectively. Inter- and intra-species differences in sequence lengths were mainly caused by variations in *CR2* (Figure 1). Each mitogenome contained the typical 13 PCGs, 22 tRNA genes, two rRNA genes, and two *CRs* (Figure 1). Overall, 28 genes were encoded on the H-strand, while the *ND6* gene and 8 *tRNAs* were encoded on the L-strand (Figure 1). The gene arrangement order of the four lark mitogenomes is the same: All are *Cytb-trnT-CR1-trnP-ND6-trnE-rCR2-trnF-rrnS*. These characteristics are similar to other lark mitogenomes published in the past, such as *A. arvensis* [26] and *Melanocorypha mongolica* [27].

Similar to other avian mitogenomes [72,73,74], the A + T content was higher than the G + C content in either the whole or every partition of mitogenomes (PCGs, *tRNAs*, *rRNAs*, and *CRs*). The highest A + T content was found in *CR2*, ranging from 67.1% in *A. cheleensis* to 79.2% in *E. alpestris*. The composition skew showed that the AT skew of the whole mitogenome was positive, while the GC skew was negative, indicating the presence of more As than Ts and more Cs than Gs, respectively (Table S5). For different partitions of mitogenomes, PCGs showed slight A skew and obvious C skew, *rRNAs* showed moderate A skew and slight C skew, *tRNAs* showed slight A/G skew, *CR1* showed slight T skew and obvious C-skew, and *CR2* showed slight A skew and obvious C skew. Among the three codon positions, an obvious T skew was recovered at the second position, while the most significant A skew was found at the third position. Although all three codon positions showed C skew, the degree of bias increased gradually from first to third (Table S5).

The RSCU results of 13 PCGs indicated that codons ending with A and C were more frequent than those ending with U and G (Figure S2). This observation was consistent with the results of nucleotide skews and may be a result of the nucleotide bias present in the mitogenome [75,76]. The start/stop codons of 13 PCGs were conserved among two species, except for the stop codon of Cytb. All PCGs were initiated with ATG and terminated with one of the three types of stop codons including the standard (TAA, TAG), the non-standard (AGG, AGA), and the incomplete (TA, T) (Table S6). All tRNAs were predicted to form the typical cloverleaf structure including the amino acid acceptor arm, anticodon arm, dihydrouracil arm and TψC arm, except trnS1 (AGY), which had lost the dihydrouracil arm in most metazoan mitogenomes (such as *E. alpestris* in Figure S3).

### 3.2. Phylogenetic Analyses

**Figure 2:**
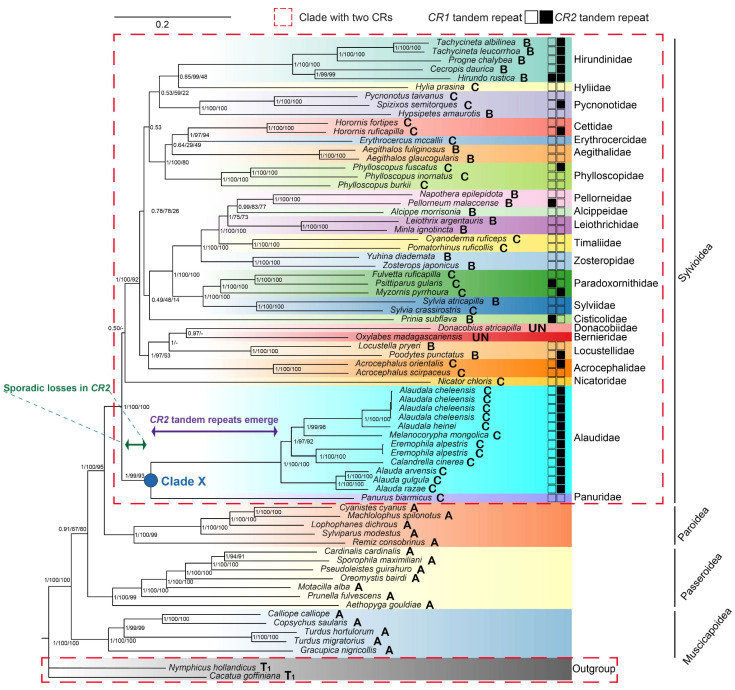
Inferred phylogenetic relationships based on mitogenomes using BI and ML. The topological structure obtained from BI is shown. Node support values are listed at nodes in the order PP/SH-aLRT/BP, and - indicates topological conflicts. The species enclosed in red dashed lines has two *CRs*. The gene order (A, B, C, T1, UN = unknown) corresponding to Table S3 for each species is labeled after the species name (Specific gene order see Section 3.4 below). The left rectangle and the right rectangle following the species name represent the tandem repeats of *CR1* and *CR2*, respectively. Unfilled indicates the absence of repeat, while filled indicates the presence of repeat. The green and purple bidirectional arrows represent the time of *CR2* sporadic losses and the emergence of *CR2* tandem repeats, respectively. Branch lengths of the outgroup have been shortened.

BI and ML phylogenetic trees showed nearly identical topologies in which 69 Passerida mitogenomes diverged into four major clades (Muscicapoidea, Passeroidea, Paroidea, and Sylvioidea) with maximal nodal support (PP ≥ 0.91, SH-aLRT ≥ 87, and BP ≥ 80, Figure 2). In Sylvioidea, Alaudidae is sister to Panuridae (PP = 1, SH-aLRT = 99, and BP = 93,) and both families formed a root clade X. The remaining families, including Nicatoridae, formed a separate cluster with less support. In Alaudidae, three *Alauda* species formed a clade sister to a clade containing all the remaining species in this family. In the latter clade, the clade containing *E. alpestris* and *C. cinerea* is sister to the clade containing *M. mongolica* and *Alaudala* genus. All nodes within Alaudidae are well supported (PP = 1, SH-aLRT ≥ 96.7, and BP ≥ 92).

The mt gene order and tandem repeats in *CRs* were mapped onto the phylogenetic tree (Figure 2). All Sylvioidea species as well as two outgroup species have duplicated *CRs* with the gene order B, C, or T1. Tandem repeats in *CR2* are widely present in Alaudidae and Hirundinidae but only occur in individual species of a few other families in Sylvioidea (Figure 2, Table S2). The mt gene order C with *rCR2* occurred in the clade X of Alaudidae and Paridae, in which *CR2* tandem repeats only appeared in the *rCR2* of all Alaudidae.

### 3.3. CR Analysis

**Figure 3:**
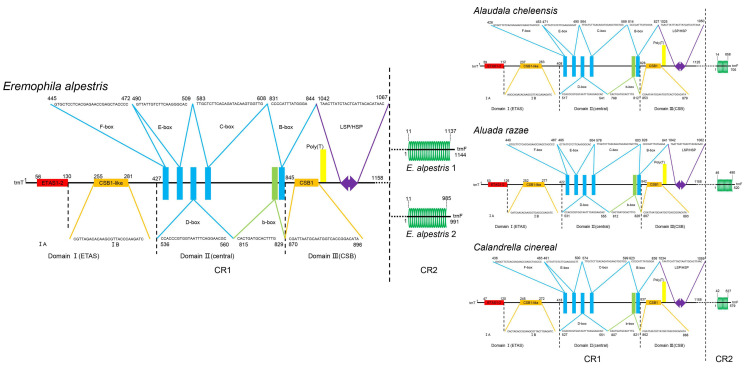
The molecular organization of the *CRs* in new mitogenomes. The structures of CR1 and CR2 are respectively displayed on the left and right sides of the vertical dashed line. Different colors are used to represent distinct motifs. Green circles represent tandem repeat (Each circle represents a repeat unit.) The size of the gene is not scaled.

The *CR1s* could be divided into three domains [26] (Figure 3). ETAS1-2 and CSB1-like elements were distributed in Domain I. There were six conserved sequence elements: F-boxes, E-boxes, D-boxes, C-boxes, b-boxes, and B-boxes in Domain II. A LSP/HSP region and a CSB1 conserved element with a downstream poly-T were distributed in Domain III. However, these conservative sequence elements of *CR1* were not detected in *CR2*. Instead, only tandem repeats were found (Figure 3).

**Figure 4:**
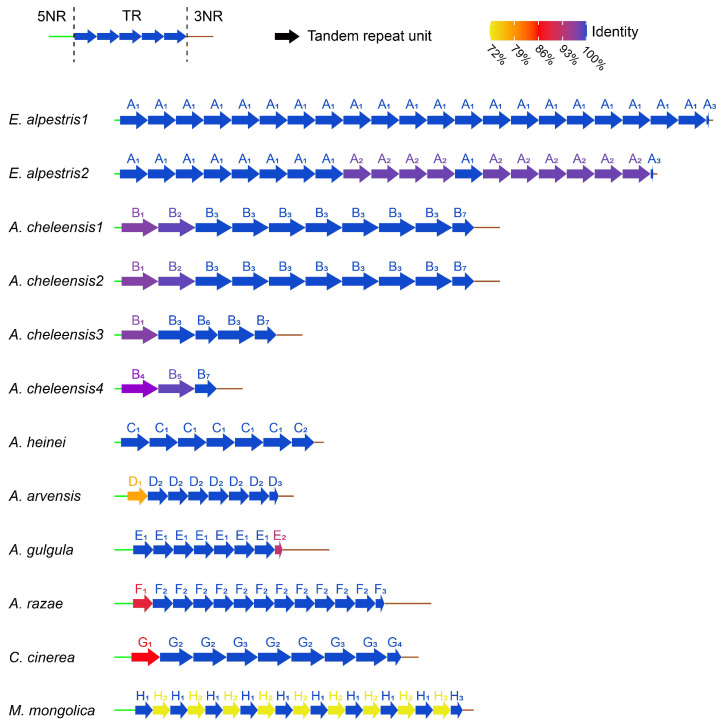
The structure of *rCR2* in 12 larks. The green horizontal line represents the sequence of 5NR, the brown horizontal line represents the sequence of 3NR, and the arrow represents each tandem repeat unit. The color of the arrow indicates the identity between the sequence of this repeat unit and the repeat unit consensus sequence within species. The lengths of 5NR, TR, 3NR, and each repeat unit are scaled according sequence lengths.

**Figure 5:**
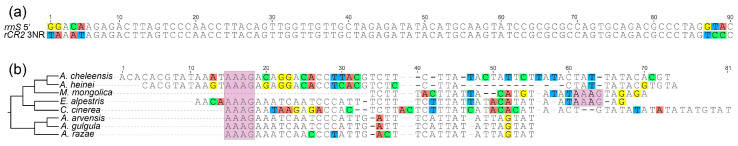
(**a**) Alignment among 3′ NR of *rCR2* and 5′ *rrnS* in *A. gulgula*. (**b**) Alignment among repeat unit in *rCR2* of eight different larks. The sequences of all the larks were aligned using consensus sequences, except for *M. mongolica*, which was aligned using the H_1_. Inconsistent loci among sequences were marked by a colored background.

The structure of *rCR2* could be divided into three regions (Table S7, Figure 4): 5′ non-repeat region (5NR), tandem repeat (TR), and 3′ non-repeat region (3NR). Both non-repeat regions showed intra-species conservation and inter-species variation (Table S7, Figure 4). To determine the origin of tandem repeats in *rCR2*, we compared the sequences of seven patterns, including 5NR, TR unit, 3NR, 5NR + TR unit, TR unit + 3NR, 5NR + TR unit + 3NR, and 5NR + 3NR, with any partial sequences on each mitogenome. Apart from the 3NR of *rCR2* aligning with 100% similarity to the *rrnS* 5′ in *Alauda gulgula* (Figure 5a), we did not find any homologous regions in the entire mitogenome. This suggests that *CR2* has undergone sporadic deletions.

In TR, the copy numbers of repeat units varied both inter- and intra-species. Sequences of repeat units differed greatly in length and similarity inter-species but were conserved intra-species (Table 1, Tables S7 and S8 and Figure 4). It should be noted that the initial repeat units (D_1_, F_1_, and G_1_) of *A. arvensis*, *A. razae*, and *C. cinerea* were significantly different from the other repeat units within species, while there were no point mutations among the other repeat units within species (Table 1, Figure 4). Additionally, in the *rCR2* of *M. mongolica*, there were nine mutation differences between two types of smaller repeat units (H_1_ and H_2_), which combined to form a new repeat unit (H_1_ + H_2_). The similarity between the newly formed combined units was 100% (Table 1).

We manually aligned the consensus sequences of repeat units for different species and calculated the pairwise similarity (Figure 5b, Table S8). We found that these sequences are homologous and exhibit greater similarity among species that are closely related. In the alignment, *Alaudala* genus species and *C. cinerea* exhibited lower similarity with other larks due to the presence of some TATA motifs. Repeat units of *M. mongolica* are more similar to the latter half of repeat unit of *E. alpestris*. It is worth noting that the AAAG motif exists in all larks and appears twice in *E. alpestris*. Combined with the special repeat pattern ([H_1_ + H_2_]n) found in *M. mongolica*, it is speculated that the repeat unit of the *E. alpestris* has evolved through the fusion of two ancestral single units.

**Figure 6:**
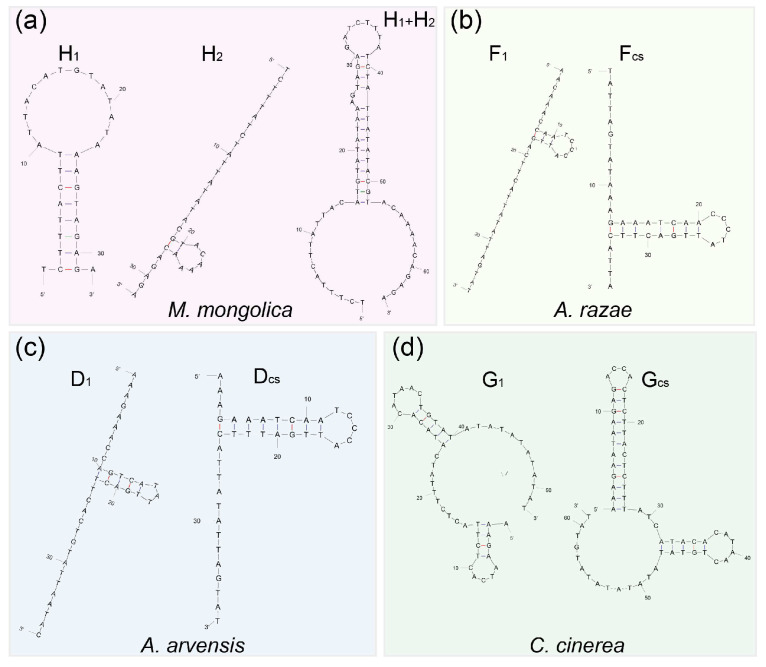
(**a**) The secondary structures of two types of units (H_1_ and H_2_) and their combination (H_1_ + H_2_) in *M. mongolica*. (**b**–**d**) The secondary structures are predicted, respectively, by the initial repeat unit and the consensus sequences of subsequent repeat units.

We examined the secondary structures of one and two units in *CR2* tandem repeats in eight different larks (Table 1, Figure S4). Nearly all reconstructed secondary structures of repeat units consisted of stems and loops and formed multiple or extensive stem-and-loop structures. In contrast to one repeat unit, the secondary structures of combinations of two repeat units were more complex, with higher free energy values (Table 1, Figure S4). Interestingly, the repeat unit H_2_ of *M. mongolica* cannot spontaneously form a secondary structure (0.1 kcal M-1), but it can form a secondary structure with higher free energy by combining with H_1_ into a repeat unit (H_1_ + H_2_) (Figure 6a). Additionally, the initial repeat unit of *A. arvensis*, *A. razae*, and *C. cinerea* cannot form a stable secondary structure, and its free energy is lower compared to that of the subsequent repeat units (Table 1, Figure 6b–d), suggesting that they may have been eliminated during the evolution of tandem repeats.

### 3.4. Gene Rearrangement

**Figure 7:**
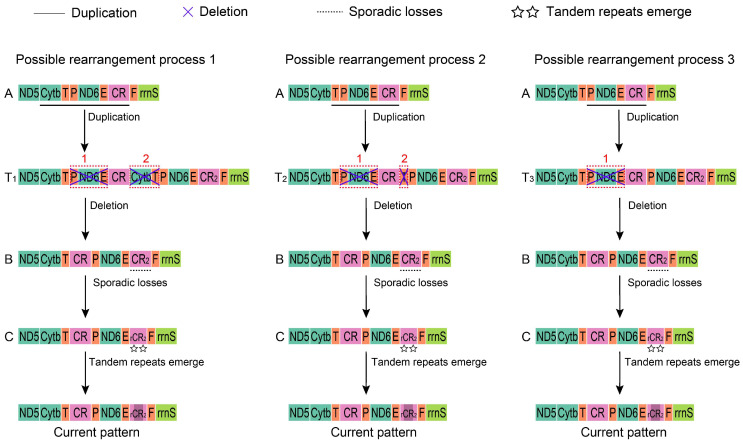
Three possible rearrangement processes of Alaudidae mitogenomes. The size of the gene is not scaled. The black horizontal line indicates duplications of gene blocks. The blue cross mark indicates the random loss of the duplicated genes, and the red numbers above them indicate the times lost. The black horizontal dashed line indicates sporadic losses in *CR2*. The double stars indicate emergence of tandem repeats. Different types of genes are labeled with different colors. The black block in *rCR2* represents tandem repeats. A, B, and C represent different mt gene orders. T1, T2, and T3 represent transitional states of gene rearrangement.

Based on the TDRL model and previous research, we speculated on three possible processes of mt gene arrangement for these Alaudidae species starting from the avian ancestral gene order (type A) [10,11,12,13] (Figure 7). In process I, the *Cytb-trnT-trnP-ND6-trnE-CR* fragment in the mt gene order A was first duplicated, followed by the loss of two small fragments (*trnP-ND6-trnE* and *Cytb-trnT*). Then, as indel events and mutations continuously accumulated in completed *CR2*, the mt gene order B transitioned to C, with tandem repeats emerging last in *rCR2*. The shorter fragment of *trnT-trnP-ND6-trnE-CR* duplicated first, and then, two losses of *trnP1-ND6-trnE and trnT* occurred in process II, while in process III, the shortest fragment of *trnP-ND6-trnE-CR* was firstly duplicated, followed by a loss of *trnP-ND6-trnE*. The rest of the rearrangement in processes II and III were the same as in process I, leading to the final observed mt gene order.

## 4. Discussion

### 4.1. Phylogenetic Relationships

Sparse sampling in phylogenetic studies of Sylvioidea species can introduce bias. Some molecular phylogenetic studies have neglected to include species from Panuridae and Nicatoridae, leading to the placement of Alaudidae at the edge of Sylvioidea [11,28,29,30,31,32,33,34]. However, when including either Nicatoridae species [35,36] or Panuridae [37,38] or both [39,40,41,42,43,44,45], Alaudidae and Panuridae were found to cluster together and form a sister clade to the rest of the birds in Sylvioidea. In this study, the mitochondrial genomes provided further evidence for the sister relationship between Alaudidae and monotypic family Panuridae, confirming their basal position within Sylvioidea (Figure 2). It is worth noting that the location of Nicatoridae was uncertain due to low nodal support. This might be resolved with large data sets, as indicated by previous studies [44,45]. The phylogenetic relationships within Alaudidae were well supported and consistent with both phylogeny using multi-locus and SNP datasets [23,24].

### 4.2. Evolutionary Progression of Mt Gene Arrangement in Alaudidae

The study by Mackiewicz [11] suggested that the arrangement of the mt gene in Sylvioidea may have been the result of recent independent rearrangements or inheritance from an early Psittaciformes ancestor. The TDRL model, which accounts for intermediate gene orders and residual repetitive genes, is considered the most plausible explanation for these rearrangements [10,11,12,13]. In relation to Alaudidae, we speculated on three possible processes of mt gene rearrangement (Figure 7). The mt gene order with three types of duplication (T1, T2, and T3, Figure 7) was similar to those found in other birds, such as T1 in *Ardea cinerea* [4] and *Grus virgo* [6]; T2 in *Bubo blakistoni* [3] and *Diomedea melanophris* [7]; and T3 in *Crypturellus tataupa* and *Rhea americana* [10]. If the mt gene arrangement of Alaudidae was a recent occurrence within Sylvioidea, all three possible rearrangement processes of Alaudidae mitogenomes would be possible. Process III was the most parsimonious compared to processes I and II. However, if the mt gene order in Sylvioidea (Passeriformes) was inherited from ancestor Psittaciformes, then process I becomes the most likely explanation for the observed gene order given the specific mt gene duplication (T1) found in Psittaciformes.

A special case is observed in *A. gulgula*, where the *rCR2* 3NR and *rrnS* 5′end show a 100% similarity across 81 base pairs (Figure 5a), which is hard to explain with the TDRL model. An alternative explanation is the occurrence of a recent recombination event. While avian mtDNA genetic variation is generally thought to be minimally affected by recombination [77], there is experimental and circumstantial evidence suggesting the existence of recombination in animal mtDNA [14,78]. Two recombination models explained the origin of this sequence: One involves direct inter-molecular insertion induced by the mini-circles excised from one mtDNA molecule [79], while the other involves illegitimate elongation induced by the stem-loop structures in tRNAs or some non-replication origins [80]. Further investigation and empirical evidence are needed to determine the precise mechanism underlying this phenomenon.

Additionally, all observed mt gene orders in clade X of Alaudidae and Panuridae have been classified as type C with *rCR2* (Figure 2 and Figure 6). This suggests that *CR2* duplications and sporadic losses occurred prior to the divergence between Alaudidae and Panuridae, and this trait could be a synapomorphy of clade X (Figure 2). Taking into account that Alaudidae diverged approximately 20 million years ago [23,24,44,45], it is plausible that the type C with *rCR2* arrangement has existed for over 20 million years. Certainly, sequencing more complete mitogenomes from other larks could further highlight the evolutionary progression of mt gene arrangement in Alaudidae.

### 4.3. Evolutionary Progression of rCR2 in Alaudidae

Unlike other birds with *CR2* sharing similarity with *CR1* [2,3,4,5,6,7,8,9,10,11,12], the *CR2* of these larks has lost homology with *CR1* due to severe degradation and the presence of tandem repeats. We established the homology between these tandem repeats among larks, suggesting that their ancestor’s *CR2* already possessed tandem repeats (Figure 2). However, it is more likely that these repeats originated from an ancestor of Alaudidae after the divergence of clade X rather than being inherited from the *CR1* (Figure 2), similar to the *CR2* tandem repeats observed in Accipitridae [81]. This is supported by the absence of repeats or homologous sequences in *CR1* of any Alaudidae species. Meanwhile, the occurrence of *CR* tandem repeats in Sylvioidea species was infrequent (Figure 2, Table S2) and showed no association with tandem repeats in *CR1* (Fisher’s exact test *p* = 0.1063). Additionally, the distribution of tandem repeats in *CR2* was random in all Sylvioidea species except for Alaudidae and Hirundinidae (Figure 2, Table S2). All of this reduced the possibility of tandem repeats in *rCR2* being inherited from *CR1* when tracing back to the common ancestor of Sylvioidea. Similarly, tandem repeats only appeared at the end of complete *CR2* that was highly homologous to *CR1* in some birds of Sylvioidea, such as *Locustella pleskei* (KY230383), *Poodytes punctatus* (KC545398), and *Acrocephalus orientalis* (NC_046418) (Figure S5).

Due to the lack of extensive recombination in avian mtDNA [77], slipped-strand mispairing is often considered the cause of the generation and evolution of tandem repeats, especially when single strands of the repeat units can form stable secondary structures [18,21,22]. This mechanism is well supported in these mtDNAs of larks: One or two repeat units could form stable secondary structures with multiple stem-loop and high free energy in all examined mtDNAs. In addition, the tandem repeats of these larks should originate from a common ancestor, but the paralogs (within species) of these repeat units are more similar than the orthologs (between species), which can be explained by the turnover model [20] or gene conversion [82]. The turnover model refers to the phenomenon that there was a turnover of repeat units within a tandem repeat, with new unit types arising through mutations and increasing in frequency through duplications and with the possibility of some types being lost through deletions and genetic drift [20,83]. This process is closely related to slipped-strand mispairing, and units capable of forming stable secondary structures will have an advantage in evolution [16,22]. Gene conversion refers to homologous recombination, where two duplicate genes exchange sequences between two molecules or within a single molecule [82]. Although both processes can make paralogs more similar than orthologs, considering that (1) the repeat units of these larks can form stable secondary structures, (2) the fusion of two units into one unit in *E. alpestris*, (3) the special arrangement of repeat units and comparison of secondary structures between repeat units in the *M. mongolica*, and (4) that the initial unit of tandem repeats has a less stable secondary structure (indicated by lower free energy) compared to the subsequent units in *A. arvensis*, *A. razae*, and *C. cinerea*, the DNA turnover model appears to be more suitable for describing the evolution of tandem repeats of larks.

## 5. Conclusions

In this study, mitogenomes of *Alaudala cheleensis*, *Eremophila alpestris*, *Alauda razae*, and *Calandrella cinerea* were characterized. Similar to other larks, these mitogenomes have a higher AT content (highest in *CR2*) and a tendency toward A skew and C skew. Codons ending with A and C were more frequent than those ending with U and G, and all tRNAs could be folded into classic cloverleaf structure except for trnS1 (AGY). All lark mitogenomes shared gene rearrangements of *Cytb-trnT-CR1-trnP-nad6-trnE-rCR2-trnF-rrnS* and contained tandem repeats in *rCR2*. The TDRL model can effectively explain the evolution of gene order, suggesting that this gene rearrangement could have evolved from the ancestral gene order in at least three ways. Alaudidae and Panuridae are sister groups at the base of Sylvioidea, and sporadic losses of *CR2* may occur in their common ancestor. The tandem repeats in *rCR2* are thought to have originated from an ancestor of Alaudidae rather than being inherited from *CR1* (ancestor of Sylvioidea or Psittaciformes). They are most likely generated through slipped-strand mispairing and evolve through DNA turnover. Sequencing more lark mitogenomes could provide a better understanding of the gene rearrangements and *CR2* tandem repeats of Alaudidae.

## Figures and Tables

**Table 1:** Sequence information for the haplotypes and consensus sequences of *rCR2* repeat units of eight lark species.

Species	Haplotype	Sequence (5′–3′)	Similarity among Haplotype	Free Energy (kcal/mol)	
				One Unit	Two Unit
*E. alpestris*	A_CS_	AACAAAAGAAATCAATCCCATTTCTTTCTTTATTATACATATAATAAAGAG	98.0~100.0%	−9.04	−18.26~−18.08
	A_1_	...................................................			
	A_2_	..................T................................			
	A_3_	.....----------------------------------------------			
*A. cheleensis*	B_CS_	ACACACGTATAAATAAAGACAGGACACCTT-ACGTCTTCTTATACTATTCTTATACTATTATACACGT	94.1~100.0%	−3.23~−2.63	−8.17
	B_1_	......A.....G.................-.....................................			
	B_2_	..............................T.....................................			
	B_3_	..............................-.....................................			
	B_4_	......A.....G.................-..........................G..........			
	B_5_	..............................-..........................G..........			
	B_6_	.........---------------------------................................			
	B_7_	..............................-...........--------------------------			
*A. heinei*	C_CS_	CACGTATAAGTAAAGAGAGGACACCTCACGTCTCCTTACTATTATACGTGTA	100%	−8.30~−7.54	−18.48~−17.72
	C_1_	....................................................			
	C_2_	............................................--------			
*A. arvensis*	D_CS_	AAAGAAATCAATCCCATTGATTTCATTATATTAGTAT	100.0%	−6.81~−5.93	−15.36
	D_1_	.......C..G..ATA....C....C.G.....A..C		−2.58~−2.49	
	D_2_	.....................................			
	D_3_	.................--------------------			
*A. gulgula*	E_CS_	AAAGAAATCAATCCCATTGATTTCATTATATTAGTAT	92.9%	−6.81~−5.93	−15.36
	E_1_	.....................................			
	E_2_	....G.........-----------------------			
*A. razae*	F_CS_	AAAGAAATCAACCCTATTGACTTCATTATATTAGTAT	100%	−3.10~−2.22	−7.94
	F_1_	..CA..CCA.T...-......................		−0.90~−0.10	
	F_2_	.....................................			
	F_3_	................---------------------			
*C. cinerea*	G_CS_	AAAGAATAAGAGACCACTCTTACTCTTTATCATACACATAACTGTATATATATATATGTAT	100%	−7.70~−6.82	−19.60
	G_1_	.......---------.A..CTTA..C.T.ATC.T...C.TAAC.G..........TA...		−2.92~−2.31	
	G_2_	.............................................................			
	G_3_	............................................----.............			
	G_4_	..........................			
*M. mongolica*	H_1_	TCTTTACTTATTACATGTATATAAAGTAGAGA		−2.03	
	H_2_	......TC.....T..ACG..C...AC.....		0.10	
	H_3_	......................----------			
	H_1_ + H_2_	TCTTTACTTATTACATGTATATAAAGTAGAGATCTTTATCTATTATATACGTACAAAACAGAGA	100.0%	−3.65	

The haplotypes correspond to the Table S7 column “Tandem repeats”. Dashes indicate gaps, and dots indicate identity sites. The first repeat units (D_1_, F_1_, and G_1_) of *A. arvensis*, *A. razae*, and *C. cinerea* were excluded when calculating similarity among intra-species haplotype because they may be discarded during the evolution of tandem repeats. The free energy of sequence corresponds to the secondary structure shown in Figure 5 and Figure S5.

## Data Availability

The genome sequence data that support the findings of this study are openly available in GenBank of NCBI at https://www.ncbi.nlm.nih.gov/, accessed on 24 April 2024 (Accession number: MH061200-MH061203, PP719628-PP719629).

## Supplementary Materials

The following supporting information can be downloaded at: https://www.mdpi.com/article/10.3390/life14070881/s1, Figure S1: Phylogenetic tree of several *Alaudala* species based on the *Cyt b* under three models; Figure S2: RSCU analysis of the PCGs of six mitogenomes of six species of larks; Figure S3: Secondary structures of 22 *tRNAs* of *E. alpestris* mitogenome; Figure S4: Secondary structures of one and two units; Figure S5: Alignment among *CR1* and *CR2* in some Sylvioidea species; Table S1: PCR primers used in this study for amplification and sequencing; Table S2: Published mitogenomes of Sylvioidea species; Table S3: GenBank accession numbers and gene order for the 71 sequences in this study; Table S4: The sites, subset partitions, and best models used in phylogenetic analysis; Table S5: Nucleotide composition and bias of six novel mitogenome; Table S6: Initial and terminal codons for protein-coding genes; Table S7: The structure of *rCR2* in twelve larks; Table S8: Pairwise similarity for consensus sequences of repeat unit in *CR2* repetitive sequences among eight lark species.
