# Supplementary material for: Selected Lark Mitochondrial Genomes Provide Insights into the Evolution of Second Control Region with Tandem Repeats in Alaudidae (Aves, Passeriformes)
Source: Life (Basel). 2024 Jul 15;14(7):881. doi: 10.3390/life14070881 (PMC11278119; doi:10.3390/life14070881)
Supplement: Supplementary file 1 [file life-14-00881-s001.zip › life-3046625-supplementary.pdf]

Table S1. PCR primers used in this study for amplification and sequencing.

No.	Primer	Nucleotide sequence (5'-3')	Binding Site(bp)
1	L1267	ACACAAAGCATGGCACTGAA[a]<br>ATTCAAAGCATGGCGCTGAA[b]	*trnF* (14)
	H2294	CTTTCAGGTGTAAGCTGARTGCTT[ab]	*trnV* (1091)
2	L2258	CGTAACAAGGTAAGTGTACCGGAAGG[ab]	*rrnS* (1009)
	H6681	GGTATAGGGTGCCGATGTCTTTGTG[a]<br>GGTAGAGTGTGCCGATGTCTTTGTG[b]	*COX1* (5455)
3	L6615	CCTCTGTAAAAAGGACTACAGCC[ab]	*trnY* (5343)
	H9233	AAGAAGCTTAGGTTCATGGTCAGG[ab]	*ATP6* (8018)
4	41f	ATCGTAGTGGAAGCCGCTC[a]<br>ATCGTAGTAGAAGCCACCC[b]	*COX2* (7710)
	41r	GAGCAGGGCGATTTCTAGGT[a]<br>AAGTAGGGCAATTTCTAGGT[b]	*ND3* (9762)
5	42f	CCCTAGGATCAGCCCGACTC[ab]	*ND3* (9674)
	42r	GTTCTTGTTGGTTGGTCCTCC[ab]	*TrnS1* (11730)
6	L51	CCAGCATGCAAGTGTAGTTTAAA[a]<br>CCCACATGCAAGTATAGTTTAAA[b]	*ND4* (11628)
	H51	TGATCCTGAGTGCAGGGTGGTG[a]<br>TAGCCCTGAGTGAAGGGTTGTG[b]	*ND5* (13596)
7	L54	ACAGCCGGGTTTCTCATCAC[a]<br>ACAGCCGGATTCTTCATCAC[b]	*ND5* (13231)
	H54	AGGTGTAGTCTTCAATCTTTGGTTT[a]<br>AGGTGTAGTCTTCAGTCTTTGGTTT[b]	*trnT* (14875)
8	L62	TAGGGTTCGCCCTCCTACTC[a]<br>TAGGATTCGCCCTCCTGC[b]	*Cytb* (14363)
	H62	TGGGAGTTGGTGATGAGGGT[ab]	*trnP* (16051)
9	L61	CACTTCACCTCGGAAACGGATAATG[ab]	*CR* (15173)
	H61	GTGCCTGATGCCTGCTCCTA[ab]	*rrnS* (190)

The primer marked with a and b was used to amplify the mitogenome fragment of *A. cheleensis* and *E. alpestri*, respectively. The number in binding site was the position of the beginning or the end of primer according to the mitogenome of *A. cheleensis*.

Table S2. Published mitogenomes of Sylvioidea species.

Family	Species published	Accession	*CR1* TP	*CR2* TP	*CR2* integrity
Acrocephalidae	*Acrocephalus orientalis*	NC_046418	**None**	**Presence**	Complete
	Acrocephalus scirpaceus	NC_010227	**None**	**None**	Complete
Aegithalidae	*Aegithalos bonvaloti*	NC_024267	**None**	**None**	Complete
	Aegithalos caudatus	KF951088	**None**	**None**	Complete
	Aegithalos concinnus	KF951091	**None**	**None**	Complete
	Aegithalos fuliginosus	NC_024266	**None**	**None**	Complete
	Aegithalos glaucogularis	KF951090	**None**	**None**	Complete
	Leptopoecile sophiae	MZ677204	**None**	**None**	Complete
Alaudidae	*Alauda arvensis*	NC_020425	**None**	**Presence**	Remnant
	Alauda gulgula	NC_057960	**None**	**Presence**	Remnant
	Alaudala cheleensis	This study	**None**	**Presence**	Remnant
	Eremophila alpestris	This study	**None**	**Presence**	Remnant
	Alauda razae	This study	**None**	**Presence**	Remnant
	Calandrella cinerea	This study	**None**	**Presence**	Remnant
	Melanocorypha mongolic	NC_036760	**None**	**Presence**	Remnant
Alcippeidae	*Alcippe morrisonia*	KX376475	**None**	**None**	Complete
Bernieridae	Data not available		**Unknow**	**Unknow**	
Cettiidae	*Horornis annae*	MW361243	**None**	**Presence**	Remnant
	Horornis fortipes	MK051002	**None**	**None**	Remnant
	Horornis parens	MW361244	**None**	**Presence**	Remnant
	Horornis ruficapilla	MW361247	**None**	**Presence**	Remnant
	Horornis vulcanius	NC_053103	**None**	**None**	Remnant
Cisticolidae	*Prinia subflava*	CM037599	**Presence**	**None**	Complete
Donacobiidae	Data not available		**Unknow**	**Unknow**	
Erythrocercidae	*Erythrocercus mccallii*	NC_053070	**None**	**None**	Remnant
Hirundinidae	*Cecropis daurica*	NC_024107	**None**	**Presence**	Complete
	Delichon urbicum	MN823064	**Presence**	**Presence**	Complete
	Hirundo aethiopica	NC_050293	**Presence**	**Presence**	Complete
	Hirundo albigularis	NC_050299	**None**	**Presence**	Complete
	Hirundo angolensis	NC_050287	**Presence**	**Presence**	Complete
	Hirundo atrocaerulea	NC_050278	**None**	**Presence**	Complete
	Hirundo dimidiata	MT471263	**None**	**Presence**	Complete
	Hirundo neoxena	MN834119	**None**	**Presence**	Complete
	Hirundo nigrita	NC_050291	**Presence**	**Presence**	Complete
	Hirundo rustica	NC_050297	**Presence**	**Presence**	Complete
	Hirundo smithii	MN853142	**None**	**Presence**	Complete
	Hirundo tahitica	MN833781	**None**	**Presence**	Complete
	Progne chalybea	NC_020605	**None**	**Presence**	Complete
	Riparia riparia	NC_060992	**None**	**Presence**	Complete
	Tachycineta albilinea	NC_020601	**None**	**Presence**	Complete
	Tachycineta albiventer	NC_020602	**None**	**Presence**	Complete
	Tachycineta bicolor	NC_020596	**None**	**Presence**	Complete

	Tachycineta cyaneoviridis	NC_020599	**None**	**Presence**	Complete
	Tachycineta euchrysea	NC_020598	**None**	**Presence**	Complete
	Tachycineta leucorrhoa	NC_020603	**None**	**Presence**	Complete
	Tachycineta meyeni	NC_020604	**None**	**Presence**	Complete
	Tachycineta stolzmanni	NC_020600	**None**	**Presence**	Complete
	Tachycineta thalassina	NC_020597	**None**	**Presence**	Complete
Hyliidae	*Hylia prasina*	NC_051033	**None**	**None**	Remnant
Leiothrichidae	*Actinodura cyanouroptera*	NC_045387	**None**	**None**	Complete
	Argya affinis	MN848144	**None**	**None**	Complete
	Garrulax albogularis	NC_037464	**None**	**None**	Complete
	Garrulax canorus	KT633399	**None**	**None**	Complete
	Garrulax chinensis	NC_060673	**None**	**None**	Complete
	Garrulax courtoisi	NC_065197	**None**	**None**	Complete
	Garrulax formosus	NC_034353	**None**	**None**	Complete
	Garrulax lanceolatus	KR818090	**None**	**None**	Complete
	Garrulax perspicillatus	NC_026068	**None**	**None**	Complete
	Garrulax poecilorhynchus	NC_028082	**None**	**None**	Complete
	Garrulax sannio	KR869824	**None**	**None**	Complete
	Heterophasia melanoleuca	MK408609	**None**	**None**	Complete
	Ianthocincla cineracea	NC_024553	**None**	**None**	Complete
	Ianthocincla maxima	MZ129308	**None**	**None**	Complete
	Ianthocincla ocellata	NC_027657	**None**	**None**	Complete
	Leiothrix argentauris	NC_015114	**None**	**None**	Complete
	Leiothrix lutea	NC_020427	**None**	**None**	Complete
	Liocichla omeiensis	KU886092	**None**	**None**	Complete
	Minla ignotincta	NC_030588	**None**	**None**	Complete
	Trochalopteron affine	NC_029402	**None**	**None**	Complete
	Trochalopteron elliotii	NC_034373	**None**	**None**	Complete
	Trochalopteron milnei	NC_041141	**None**	**None**	Complete
Locustellidae	*Locustella pleskei*	KY230383	**None**	**Presence**	Complete
	Locustella pryeri	NC_029151	**None**	**None**	Complete
	Poodytes punctatus	KC545398	**None**	**Presence**	Complete
Macrosphenidae	Data not available		**Unknow**	**Unknow**	
Modulatricidae	Data not available		**Unknow**	**Unknow**	
Nicatoridae	*Nicator chloris*	NC_053062	**None**	**None**	Remnant
Panuridae	*Panurus biarmicus*	NC_053106	**None**	**None**	Remnant
Paradoxornithidae	*Chamaea fasciata*	CM055129	**None**	**None**	Remnant
	Fulvetta cinereiceps	MG833030	**None**	**None**	Remnant
	Fulvetta ruficapilla	NC_045401	**None**	**None**	Remnant
	Fulvetta vinipectus	NC_050289	**None**	**None**	Remnant
	Myzornis pyrrhoura	NC_051886	**None**	**Presence**	Remnant
	Paradoxornis heudei	EU376027	**None**	**None**	Remnant
	Paradoxornis gularis	NC_039536	**Presence**	**None**	Remnant
	Suthora fulvifrons	NC_028436	**None**	**None**	Remnant

	Suthora conspicillata	NC_059907	**None**	**None**	Remnant
	Suthora webbiana	NC_024539	**None**	**None**	Remnant
	Suthora nipalensis	NC_028437	**None**	**None**	Remnant
Pellorneidae	*Napothera epilepidota*	NC_035626	**None**	**None**	Complete
	Pellorneum malaccense	NC_068692	**Presence**	**None**	Complete
Phylloscopidae	*Phylloscopus borealis*	NC_045526	**None**	**None**	Remnant
	Phylloscopus borealoides	MN125373	**None**	**None**	Remnant
	Phylloscopus burkii	NC_064505	**None**	**None**	Remnant
	Phylloscopus canariensis	NC_071941	**None**	**None**	Remnant
	Phylloscopus collybita	NC_060852	**None**	**None**	Remnant
	Phylloscopus coronatus	MK533705	**None**	**None**	Remnant
	Phylloscopus examinandus	NC_051526	**None**	**None**	Remnant
	Phylloscopus fuscatus	NC_046416	**None**	**Presence**	Remnant
	Phylloscopus ibericus	NC_071943	**None**	**None**	Remnant
	Phylloscopus ijimae	LC541446	**None**	**None**	Remnant
	Phylloscopus inornatus	NC_024726	**None**	**None**	Remnant
	Phylloscopus occisinensis	MK513447	**None**	**None**	Remnant
	Phylloscopus proregulus	OR030351	**None**	**Presence**	Remnant
	Phylloscopus reguloides	NC_080910	**None**	**None**	Remnant
	Phylloscopus schwarzi	MK411584	**None**	**None**	Remnant
	Phylloscopus sibilatrix	MN122829	**None**	**None**	Remnant
	Phylloscopus sindianus	NC_071942	**None**	**None**	Remnant
	Phylloscopus tenellipes	NC_045525	**None**	**None**	Remnant
	Phylloscopus trochiloides	NC_080911	**None**	**None**	Remnant
	Phylloscopus trochilus	NC_060851	**None**	**None**	Remnant
Pnoepygidae	Data not available		**Unknow**	**Unknow**	
Pycnonotidae	*Hypsipetes amaurotis*	NC_084285	**None**	**None**	Complete
	Ixos mcclellandii	NC_039396	**None**	**None**	Complete
	Pycnonotus jocosus	NC_053063	**None**	**None**	Remnant
	Pycnonotus melanicterus	NC_024730	**None**	**None**	Remnant
	Pycnonotus sinensis	NC_013838	**None**	**None**	Remnant
	Pycnonotus taivanus	NC_013483	**None**	**None**	Remnant
	Pycnonotus xanthorrhous	NC_031830	**None**	**Presence**	Remnant
	Spizixos semitorques	NC_029321	**None**	**Presence**	Remnant
Scotocercidae	Data not available		**Unknow**	**Unknow**	
Sylviidae	*Curruca curruca*	NC_046417	**None**	**None**	Complete
	Sylvia atricapilla	NC_010228	**None**	**None**	Complete
	Sylvia borin	NC_053054	**None**	**None**	Complete
	Sylvia crassirostris	NC_010229	**None**	**None**	Remnant
Timaliidae	*Cyanoderma ruficeps*	NC_030771	**None**	**None**	Remnant
	Erythrogenys gravivox	NC_040006	**None**	**None**	Remnant
	Pomatorhinus ruficollis	NC_029769	**None**	**None**	Remnant
Zosteropidae	*Yuhina diademata*	NC_029462	**None**	**None**	Complete
	Yuhina gularis	MK405666	**None**	**None**	Complete

Yuhina nigrimenta	NC_040991	**None**	**None**	Complete
Zosterops abyssinicus	KX181885	**None**	**None**	Complete
Zosterops borbonicus	MK529728	**None**	**None**	Complete
Zosterops erythropleurus	NC_027942	**None**	**None**	Complete
Zosterops japonicus	NC_059932	**None**	**None**	Complete
Zosterops lateralis	NC_029146	**None**	**None**	Complete
Zosterops pallidus	MK524996	**None**	**None**	Complete
Zosterops poliogastrus	NC_032059	**None**	**None**	Complete
Zosterops senegalensis	KX181887	**None**	**None**	Complete

CRs with tandem repeats were marked by red (considering only the cases where repeat unit > 2bp and repeat count >2); the rows with a black background represent the species selected for phylogeny analysis in this study; data from NCBI, April 2024.

Table S3. GenBank accession numbers and gene order for the 71 sequences in this study.

Family	Species	Accession No.	Gene order
Alaudidae	*Alaudala cheleensis* 1	This study	C
	Alaudala cheleensis 2	This study	C
	Eremophila alpestris 1	This study	C
	Eremophila alpestris 2	This study	C
	Alauda razae	This study	C
	Calandrella cinerea	This study	C
	Alauda arvensis	NC_020425	C
	Alauda gulgula	NC_057960	C
	Alaudala heinei	MN356181	C
	Alaudala cheleensis 3	MG681104	C
	Alaudala cheleensis 4	MW143077	C
	Melanocorypha mongolica	NC_036760	C
Acrocephalidae	*Acrocephalus orientalis*	LC532223	B
	Acrocephalus scirpaceus	NC_010227	B
Aegithalidae	*Aegithalos fuliginosus*	NC_024266	B
	Aegithalos glaucogularis	KF951090	B
Alcippeidae	*Alcippe morrisonia*	KX376475	B
Bernieridae	*Oxylabes madagascariensis*	MN356284	UN
Cettidae	*Horornis ruficapilla*	MW361247	C
	Horornis fortipes	MK051002	C
Cisticolidae	*Prinia subflava*	CM037599	B
Donacobiidae	*Donacobius atricapilla*	MN356221	UN
Erythrocercidae	*Erythrocercus mccallii*	NC_053070	C
Hirundinidae	*Cecropis daurica*	NC_024107	B
	Hirundo rustica	NC_050297	B
	Progne chalybea	NC_020605	B
	Tachycineta albilinea	NC_020601	B
	Tachycineta leucorrhoa	NC_020603	B
Hyliidae	*Hylia prasina*	NC_051033	C
Leiothrichidae	*Leiothrix argentauris*	NC_015114	B
	Minla ignotincta	NC_030588	B
Locustellidae	*Locustella pryeri*	NC_029151	B
	Poodytes punctatus	NC_029138	B
Nicatoridae	*Nicator chloris*	NC_053062	C
Panuridae	*Panu biarmicus*	NC_053106	C
Paradoxornithidae	*Fulvetta ruficapilla*	NC_045401	C
	Paradoxornis gularis	NC_039536	C
	Myzornis pyrrhoura	NC_051886	C

Pellorneidae	*Pellorneum malaccense*	NC_068692	B
	Napothera epilepidota	NC_035626	B
Phylloscopidae	*Phylloscopus inornatus*	NC_024726	C
	Phylloscopus fuscatus	NC_046416	C
	Phylloscopus burkii	NC_064505	C
Pycnonotidae	*Hypsipetes amaurotis*	NC_084285	B
	Pycnonotus taivanus	NC_013483	C
	Spizixos semitorques	NC_029321	C
Sylviidae	*Sylvia atricapilla*	NC_010228	B
	Sylvia crassirostris	NC_010229	C
Timaliidae	*Cyanoderma ruficeps*	NC_030771	C
	Pomatorhinus ruficollis	NC_029769	C
Zosteropidae	*Yuhina diademata*	NC_029462	B
	Zosterops japonicus	NC_059932	B
Paridae	*Cyanistes cyanus*	KX388472	A
	Lophophanes dichrous	KX388477	A
	Machlolophus spilonotus	KX388476	A
	Sylviparus modestus	KP642167	A
Remizidae	*Remiz consobrinus*	KC463856	A
Cardinalidae	*Cardinalis cardinalis*	KM078795	A
Fringillidae	*Oreomystis bairdi*	KM078807	A
Icteridae	*Pseudoleistes guirahuro*	JX516071	A
Motacillidae	*Motacilla alba*	KT736087	A
Nectariniidae	*Aethopyga gouldiae*	KP772257	A
Prunellidae	*Prunella fulvescens*	KY471556	A
Thraupidae	*Sporophila maximiliani*	MF327582	A
Muscicapidae	*Copsychus saularis*	KU058637	A
	Calliope calliope	OL477598	A
Sturnidae	*Sturnus nigricollis*	JQ003192	A
Turdidae	*Turdus hortulorum*	KF926987	A
	Turdus migratorius	KJ909198	A
Cacatuidae	*Cacatue goffiniana*	MT920477	T1
	Nymphicus hollandicus	MH133968	T1

The gene orders named A, B, C, T1 correspond to in Figure 6.

Table S4. The sites, subset partitions, and best models used in phylogenetic analysis.

Software	Subset	Best Model	Sites	Subset Partitions
IQ-TREE	1	GTR+G	55	ATP8(2)
	2	GTR+I+G	11455	ATP6(2), ATP6(3), ATP8(1). ATP8(3), COX1(1), COX1(2), COX1(3), COX2(1), COX2(2), COX2(3), COX3(3), ND2(1), ND2(2), ND2(3), ND3(1), ND3(2), ND4(1), ND4(2), ND4(3), ND4L(1), ND4L(2), ND4L(3), ND5(1), ND5(2), ND5(3), ND6(1), ND6(2), Cytb(1), trnG, trnV, trnR, trnK, trnD, trnM, trnT, trnS1, rrnL, rrnS
	3	SYM+I+G	148	trnL1, trnN
	4	TIM+G	172	ND6(3)
	5	TRN+G	72	trnS2
	6	TRN+I+G	211	trnI, trnL2, trnW
	7	TVM+G	116	ND3(3)
	8	TVM+I+G	2941	trnC, trnA, trnQ, trnP, trnE, trnF, trnH, trnY, ATP6(1), COX3(2), Cytb(2), Cytb(3), ND1(1), ND1(2), ND1(3), ND6(2)
	9	TVMEF+I+G	260	COX3(1)
MrBayes	1	GTR+G	227	ATP8(2), ND6(3)
	2	GTR+I+G	14723	ATP6(1), ATP6(2), ATP6(3), ATP8(1), ATP8(3), COX1(1), COX1(2), COX1(3), COX2(1), COX2(2), COX2(3), COX3(2), COX3(3), ND1(1), ND1(2), ND1(3), ND2(1), ND2(2), ND2(3), ND3(1), ND3(2), ND3(3), ND4(1), ND4(2), ND4(3), ND4L(1), ND4L(2), ND4L(3), ND5(1), ND5(2), ND5(3), ND6(1), ND6(2), Cytb(1), Cytb(2), Cytb(3), trnC, trnQ, trnA, trnP, trnS1, trnD, trnM, trnF, trnH, trnG, trnV, trnE, trnR, trnI, trnW, trnL2, trnK, trnT, trnY, rrnL, rrnS
	3	HKY+I+G	72	trnS2
	4	SYM+I+G	408	trnL1, trnN, COX3(1)

The values enclosed in parentheses following protein-coding genes indicate the positions of codons.

Table S5. Nucleotide composition and bias of six novel mitogenome.

Feature	A+T content (%)				AT-skew				GC-skew			
	E. alpestris	***A. cheleensis***	***A. razae***	***C. cinerea***	***E. alpestris***	***A. cheleensis***	***A. razae***	***C. cinerea***	***E. alpestris***	***A. cheleensis***	***A. razae***	***C. cinerea***
Mitogenome	53.7(53.5)	52.3(52.3)	52.1	52.3	0.126(0.125)	0.144(0.144)	0.125	0.121	-0.387(-0.385)	-0.398(-0.398)	-0.382	-0.38
PCG	51(51)	50.7(50.6)	50.4	50.4	0.076(0.077)	0.103(0.103)	0.073	0.073	-0.424(-0.425)	-0.436(-0.436)	-0.416	-0.418
PCG-1st	46.5(46.6)	46.7(46.7)	47.1	47	0.173(0.172)	0.182(0.182)	0.167	0.179	-0.122(-0.121)	-0.123(-0.124)	-0.116	-0.127
PCG-2nd	58.3(58.2)	58.0(57.8)	58.2	58.1	-0.376(-0.376)	-0.373(-0.373)	-0.378	-0.374	-0.382(-0.382)	-0.383(-0.382)	-0.382	-0.385
PCG-3rd	48.3(48.4)	47.6(47.4)	46	46.1	0.527(0.531)	0.606(0.606)	0.546	0.529	-0.771(-0.774)	-0.797(-0.795)	-0.737	-0.729
rRNA	52.3(52.3)	52.3(52.3)	52.6	52.4	0.237(0.236)	0.247(0.247)	0.235	0.244	-0.113(-0.112)	-0.121(-0.121)	-0.117	-0.114
tRNA	56.3(56.3)	55.3(55.5)	54.6	56.1	0.030(0.031)	0.046(0.046)	0.025	0.040	0.022(0.022)	0.006(0.007)	0.013	0.001
CR1	56.2(56.3)	55.4(55.6)	55.5	55.9	-0.068(-0.071)	-0.05(-0.049)	-0.054	-0.111	-0.354(-0.352)	-0.343(-0.344)	-0.331	-0.331
CR2	78.3(79.2)	67.1(67.1)	68.7	72.7	0.162(0.149)	0.091(0.091)	0.106	0.135	-0.468(-0.447)	-0.474(-0.474)	-0.436	-0.481
CR-domainI	54.4(54.5)	52.9(53.2)	53.1	52.9	0(-0.004)	0.028(0.023)	0.041	-0.031	-0.364(-0.361)	-0.28(-0.277)	-0.329	-0.34
CR-domainII	51.2(51.4)	52.0(52.1)	50.7	50.8	-0.187(-0.191)	-0.188(-0.187)	-0.192	-0.201	-0.176(-0.172)	-0.208(-0.213)	-0.159	-0.175
CR-domainIII	65.1(65.1)	63.7(63.7)	64.6	66.7	-0.02(-0.019)	0.027(0.027)	-0.014	-0.102	-0.668(-0.669)	-0.704(-0.704)	-0.646	-0.644

Stop codons of protein-coding genes were excluded; The values enclosed in parentheses indicate another individual of the same species.

Table S6. Initial and terminal codons for protein-coding genes.

Species	Gene												
	ATP6	*ATP8*	*COX1*	*COX2*	*COX3*	*Cytb*	*ND1*	*ND2*	*ND3*	*ND4*	*ND4L*	*ND5*	*ND6*
Eremophila alpestris 1	ATG/TAA	ATG/TAA	ATG/AGG	ATG/TAA	ATG/T	ATG/**TAA**	ATG/AGA	ATG/TA	ATG/TAA	ATG/T	ATG/TAA	ATG/**AGA**	ATG/TAG
Eremophila alpestris 2	ATG/TAA	ATG/TAA	ATG/AGG	ATG/TAA	ATG/T	ATG/**TAA**	ATG/AGA	ATG/TA	ATG/TAA	ATG/T	ATG/TAA	ATG/**AGA**	ATG/TAG
Alaudala cheleensis 1	ATG/TAA	ATG/TAA	ATG/AGG	ATG/TAA	ATG/T	ATG/**TAG**	ATG/AGA	ATG/TA	ATG/TAA	ATG/T	ATG/TAA	ATG/**AGA**	ATG/TAG
Alaudala cheleensis 2	ATG/TAA	ATG/TAA	ATG/AGG	ATG/TAA	ATG/T	ATG/**TAG**	ATG/AGA	ATG/TA	ATG/TAA	ATG/T	ATG/TAA	ATG/**AGA**	ATG/TAG
Alauda razae	ATG/TAA	ATG/TAA	ATG/AGG	ATG/TAA	ATG/T	ATG/**TAG**	ATG/AGA	ATG/TA	ATG/TAA	ATG/T	ATG/TAA	ATG/**AGG**	ATG/TAG
Calandrella cinerea	ATG/TAA	ATG/TAA	ATG/AGG	ATG/TAA	ATG/T	ATG/**TAA**	ATG/AGA	ATG/TA	ATG/TAA	ATG/T	ATG/TAA	ATG/**AGA**	ATG/TAG

Codons with inter-species variations are highlighted in bold.

Table S7. The structure of *rCR2* in twelve larks.

Species	5’ non-repeat region (5NR)	Tandem repeats (TR)	3’ non-repeat region (3NR)
E. alpestris 1	TCCAAACAAA	$A_1A_1A_1A_1A_1A_1A_1A_1A_1A_1A_1A_1A_1A_1A_1A_1A_1A_1A_1A_1A_1A_3$	CCAAAAC
E. alpestris 2	TCCAAACAAA	$A_1A_1A_1A_1A_1A_1A_1A_1A_2A_2A_2A_2A_1A_2A_2A_2A_2A_2A_2A_3$	CCAAAAC
A. cheleensis 1	TCTAAACCCCATC	$B_1B_2B_3B_3B_3B_3B_3B_3B_3B_7$	CTACCAACGCACACAACAAACAAGAAACACCCAACCCTCCACCAAAT
A. cheleensis 2	TCTAAACCCCATC	$B_1B_2B_3B_3B_3B_3B_3B_3B_3B_7$	CTACCAACGCACACAACAAACAAGAAACACCCAACCCTCCACCAAAT
A. cheleensis 3	TCTAAACCCCATC	$B_1B_3B_6B_3B_7$	CTACCAACGCACACAACAAACAAGAAACACCCAACCCTCCACCAAAT
A. cheleensis 4	TCTAAACCCCATC	$B_4B_5B_7$	CTACCAACGCACACAACAAACAAGAAACACCCAACCCTCCACCAAAT
A. heinei	TCTAAACCCCCG	$C_1C_1C_1C_1C_1C_1C_1C_2$	CCTTTCCCCCATCAAAC
A. arvensis	TACACCTCACACCGACACAGACCA	$D_1D_2D_2D_2D_2D_2D_2D_3$	CCCCCATAAAAATGACCAAACCCAAAT
A. gulgula	TTCTCCCCACTCCACCCTATACCGACACAAACCA	$E_1E_1E_1E_1E_1E_1E_2$	TAAATAGAGACTTAGTCCCAACCTTACAGTTGGTTGTTGCTAGAGATATACATGCAAGTATCCGCGCGCCAGTGCAGACGCCCTA
A. razae	TCCCCATCCACCACCCCACAAAAACGCAAGC	$F_1F_2F_2F_2F_2F_2F_2F_2F_2F_2F_2F_2F_3$	ACCCCCCCCCATGAAACAGCCAAACCCAAAT
C. cinerea	TCCCATTACCATTCTTACCATGTATGCAAAGAAC	$G_1G_2G_2G_3G_2G_2G_3G_3G_4$	CTACCACATACAACTATAAAGAATAAAAGAAACCGACCACACCAACCCAAAC
M. mongolica	TTCAAACCAACCCACCACACAAAACAAACTTATCCACT	$H_1H_2H_1H_2H_1H_2H_1H_2H_1H_2H_1H_2H_1H_2H_1H_2H_1H_2H_3$	GGAATAAAGACCCATCAAAC

The numbered letters in TR correspond to the haplotype in Table 1 and Figure 4

Table S8. Pairwise similarity for consensus sequences of repeat unit in *CR2* repetitive sequences among eight lark species.

Similarity\gaps	*A. cheleensis*	*A. heinei*	*M. mongolica*	*E. alpestris*	*C. cinerea*	*A. razae*	*A. arvensis*	*A. gulgula*
A. cheleensis		21.7%	14.3%	50.0%	23.8%	25.0%	25.0%	25.0%
A. heinei	90.6%		15.0%	8.7%	32.3%	20.9%	25.0%	25.0%
M. mongolica	76.2%	80.0%		10.7%	39.0%	13.0%	8.7%	8.7%
E. alpestris	45.6%	65.2%	78.6%		16.7%	14.3%	14.3%	14.3%
C. cinerea	63.5%	54.8%	58.5%	66.7%		44.4%	39.3%	39.3%
A. razae	56.2%	55.8%	73.9%	69.0%	49.2%		0%	0%
A. arvensis	56.2%	54.5%	73.9%	76.2%	45.9%	91.9%		0%
A. gulgula	56.2%	54.5%	73.9%	76.2%	45.9%	91.9%	100%	

Similarity was at lower diagonal and gaps at upper diagonal; sequences of each species were represented by consensus sequences in Table 4; the similarity between *A. heinei* and *A. cheleensis* (90.6%) was calculated after removing an indel sequence, with the original value being 70.3%.

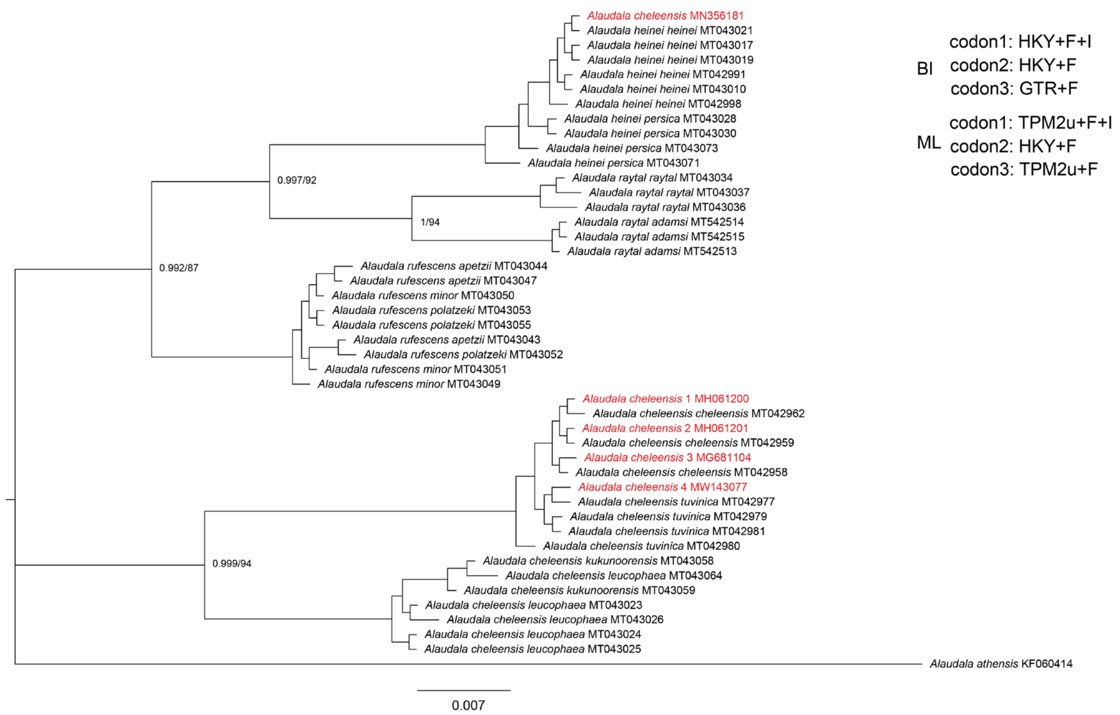

Figure S1. Phylogenetic tree of several *Alaudala* species based on the *Cytb* under three models. The NCBI accession number for each sequence is listed after the taxonomic name and the species in this study are highlighted in red. Phylogenetic analysis methods are the same as in section 2.4 of this study.

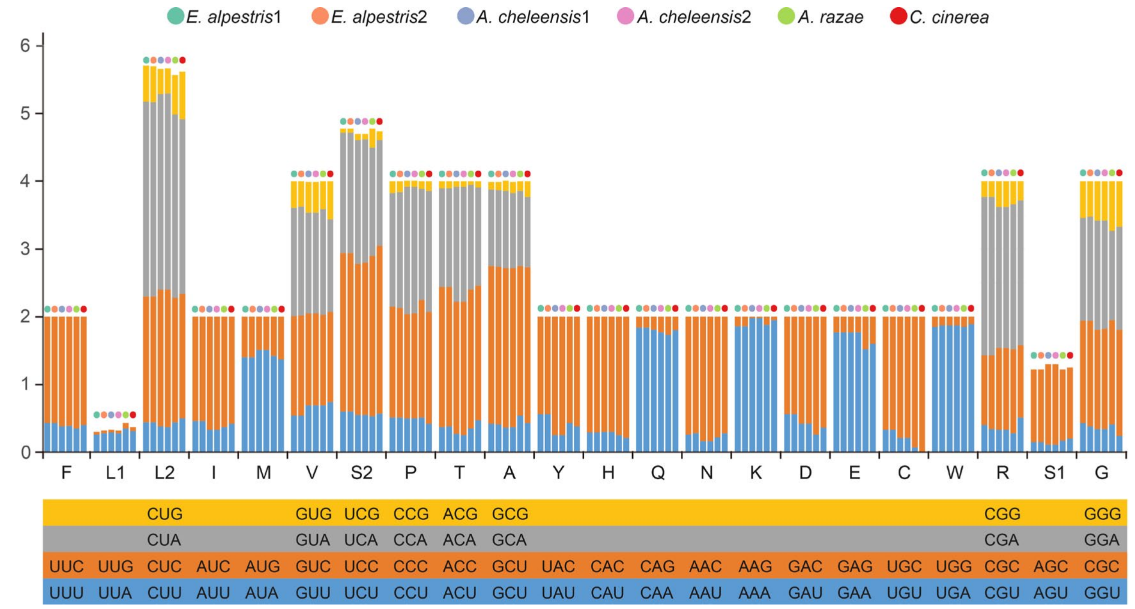

Figure S2. RSCU analysis of the PCGs of six mitogenomes of six species of larks.

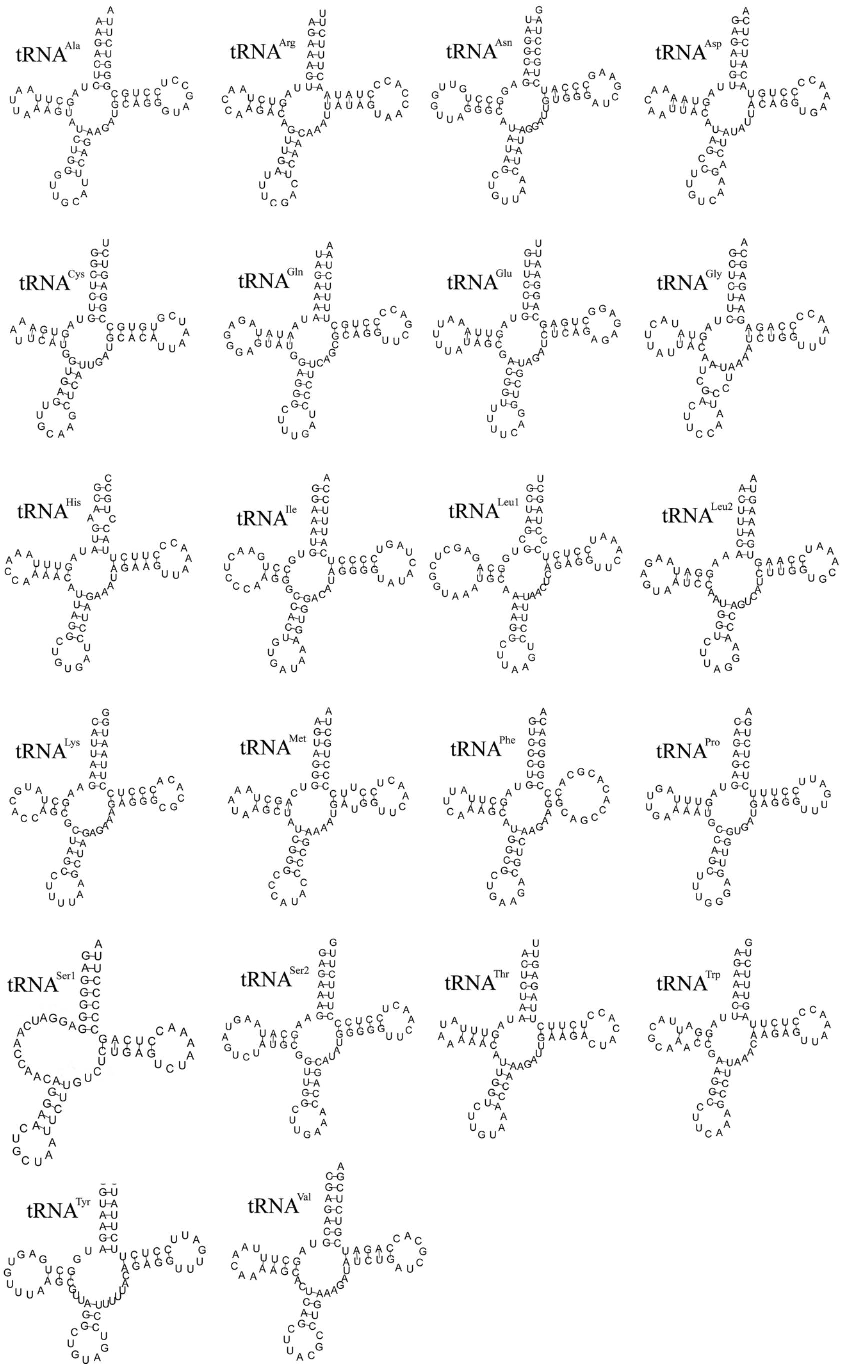

Figure S3. Secondary structures of 22 tRNA of *E. alpestris* mitogenome. Arms of the tRNAs (clockwise from the top) are the amino acid acceptor (AA) arm, the TyC (T) arm, the anticodon (AC) arm, and the dihydrouridine (DHU) arm.

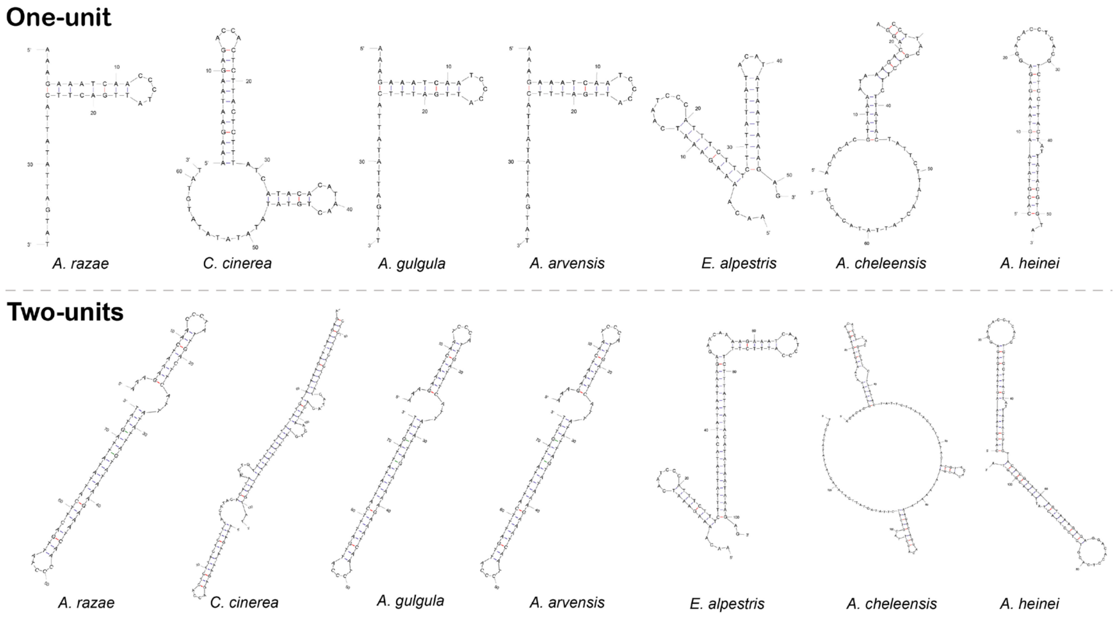

Figure S4. Secondary structures of one and two units. All were built with consensus sequences in Table 4.

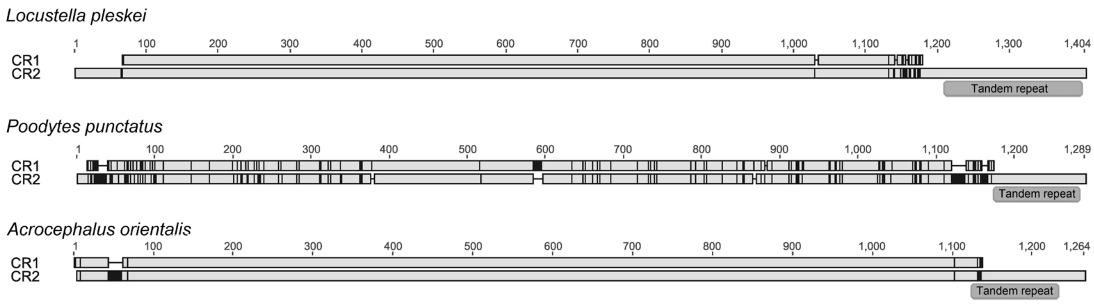

Figure S5. Alignment among *CR1* and *CR2* in some Sylvioidea species. Differences between sequences are marked by black vertical lines.
